# Artificial Intelligence Applications in Interventional Radiology

**DOI:** 10.3390/jpm15120569

**Published:** 2025-11-28

**Authors:** Carolina Lanza, Salvatore Alessio Angileri, Serena Carriero, Sonia Triggiani, Velio Ascenti, Simone Raul Mortellaro, Marco Ginolfi, Alessia Leo, Francesca Arnone, Pierluca Torcia, Pierpaolo Biondetti, Anna Maria Ierardi, Gianpaolo Carrafiello

**Affiliations:** 1 Department of Health Services, Diagnostic and Interventional Radiology, Foundation IRCCS Cà Granda-Ospedale Maggiore Policlinico, 20122 Milan, Italy; 2 Postgraduate School of Diagnostic and Interventional Radiology, University of Milan, 20122 Milan, Italy; 3 Department of Oncoematology, University of Milan, Via Festa del Perdono 7, 20122 Milan, Italy

**Keywords:** artificial intelligence (AI), interventional radiology (IR), robotics, virtual reality (VR), radiomics, three-dimensional (3D) modeling

## Abstract

This review is a brief overview of the current status and the potential role of artificial intelligence (AI) in interventional radiology (IR). The literature published in the last decades was reviewed and the technical developments in terms of radiomics, virtual reality, robotics, fusion imaging, cone-beam computed tomography (CBCT) and Imaging Guidance Software were analyzed. The evidence shows that AI significatively improves pre-procedural planning, intra-procedural navigation, and post-procedural assessment. Radiomics extracts features from optical images of personalized treatment strategies. Virtual reality offers innovative tools especially for training and procedural simulation. Robotic systems, combined with AI, could enhance precision and reproducibility of IR procedures while reducing operator exposure to X-ray. Fusion imaging and CBCT, augmented by AI software, improve real-time guidance and procedural outcomes.

## 1. Introduction

The term “artificial intelligence” (AI) refers to computational algorithms capable of performing tasks that typically require human intelligence, with partial or complete autonomy, to generate useful outputs from specific inputs [1,2].

AI represents an umbrella concept encompassing machine learning (ML) and deep learning (DL). Machine learning relies on so-called “backward training” methods, in which computer systems iteratively identify and learn specific pathological features from training datasets. A common implementation of ML involves the use of artificial neural networks (ANNs) [3,4].

A convolutional neural network (CNN) is a specialized type of deep ANN particularly suited for image analysis. CNNs are inspired by the connectivity patterns of neurons in the visual cortex, which process visual information through receptive fields and transmit these data to deeper network layers. Similar to ANNs, CNNs consist of input, output, and multiple hidden layers [5].

Despite its inherently technology-driven nature and reliance on image guidance, the application of AI in interventional radiology (IR) remains relatively underdeveloped compared to diagnostic radiology. A major contributing factor is the limited availability and sharing of large-scale datasets. Furthermore, IR procedures typically generate a lower volume of radiological images per patient and per unit time than those in diagnostic radiology. Nevertheless, the imaging data produced in IR are often smaller in size and primarily two-dimensional, which, in principle, may facilitate their processing by current AI algorithms [6]. Another major limitation arises from the intrinsic heterogeneity of interventional procedures: no two patients or interventions are identical. Variations in anatomy, body habitus, lesion characteristics, and procedural techniques often necessitate real-time adjustments, even when standardized protocols are available. This high degree of procedural individuality complicates the development of reproducible datasets and hinders the standardization required for robust AI model training. A further key challenge in the development of AI algorithms for IR is the widespread use of highly variable image acquisition protocols, operator-dependent device preferences, and the essential need for tactile feedback during procedures [7].

The application of AI in IR can be categorized into pre-, peri- and post-procedural uses [8,9]. Several tools are currently available for application of AI, including among others, radiomics, virtual reality (VR) [10], and three-dimensional (3D) simulators [11,12].

In pre-procedural assessment, AI can assist in the prescreening of patient records, enabling selection through ML and deep learning DL-based predictive models that stratify patients into likely responders and non-responders [13]. Furthermore, the integration of AI algorithms with molecular data holds promise for enhancing diagnostic accuracy and improving prognostic evaluations [14,15]. Additionally, pre-procedural virtual simulations can provide patients with an immersive preview of their upcoming intervention and enhance visualization of complex anatomical structures [16]. These technologies also offer novel approaches for education and procedural training [8,17,18]. A summary of these pre-procedural AI applications is provided in Table 1.

Intra-procedural applications of AI include the reduction in radiation exposure, enhancement of procedural precision through the use of navigation software, and the integration of imaging fusion techniques [1,8,19,20,21]. Table 2 outlines the main intra-procedural applications of AI in interventional radiology.

In the post-procedural setting, AI algorithms are expected to play a predictive role by quantifying residual disease and providing prognostic information, thereby supporting the development of personalized follow-up strategies [22,23,24]. Furthermore, AI can contribute to the evaluation of technical success and facilitate the early detection of complications [8,18].

This narrative review aims to provide a comprehensive overview of the current applications of AI in IR, with a particular focus on its role across the pre-, intra-, and post-procedural phases. In addition, it critically examines the existing limitations, technical challenges, and future directions necessary for the safe and effective integration of AI into routine clinical practice.

## 2. Pre-Procedural Applications of AI in IR

### 2.1. Radiomics: Principles and Clinical Applications in IR

Radiomics is defined as the process of converting medical images into high-dimensional quantitative data, enabling the extraction of imaging features that are imperceptible to the human eye and reflect the underlying ultrastructural characteristics of tissues. This noninvasive approach allows the derivation of detailed information from imaging data, potentially improving diagnostic accuracy and supporting personalized treatment planning [15,25,26,27].

**Figure 1:**
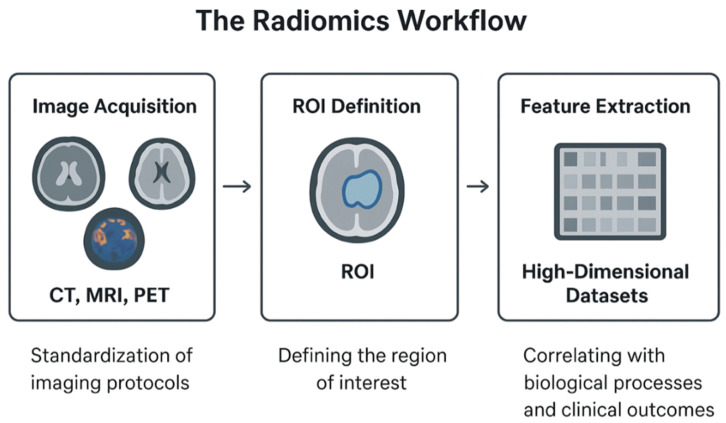
The radiomics workflow including the image acquisition, the definition of ROI, and feature extraction.

The radiomics workflow begins with the acquisition of medical images using modalities such as CT, MRI, or PET, where the standardization of imaging protocols is essential to ensure reproducibility and comparability across institutions [15,26,28,29]. Following acquisition, the region of interest (ROI) is defined, allowing quantitative descriptors to be extracted from the selected area [15,26]. These descriptors often include texture and intensity features that capture the ultrastructural architecture of tissues [30]. The extracted features are subsequently represented in high-dimensional datasets that can be correlated with underlying biological processes and clinical outcomes [15,26,31], (Figure 1).

As imaging biomarkers, these extracted features—including shape, size, and intensity metrics—can assist in diagnosis, prognosis, and treatment planning. Originally developed within the field of oncology, radiomics has since expanded to encompass multiple clinical domains.

Recent evidence indicates that radiomics holds considerable promise in the pre-procedural phase of IR, particularly for patient stratification and treatment planning [32]. For example, radiomics applied to pre-procedural CT imaging has been employed to predict survival, hepatic encephalopathy, and clinical response following trans jugular intrahepatic portosystemic shunt (TIPS) creation [33]. Similarly, MRI-based radiomics prior to ablation has demonstrated predictive value for pathological response in hepatocellular carcinoma (HCC) patients undergoing transplantation, particularly when combined with clinical variables [34]. A meta-analysis further reinforced the role of radiomics in forecasting microvascular invasion (MVI) in HCC [35].

Different studies have assessed the role of radiomics in determining the prediction of success in locoregional therapies for liver diseases including both primary and secondary lesions. He et al. [36] developed and validated a novel prognostic nomogram to evaluate the survival benefit of HCC patients receiving postoperative adjuvant trans arterial chemoembolization (PA-TACE).

Yang et al. [37] developed a model based on whole-liver radiomics features of pre-treatment enhanced MRI for predicting the prognosis of HCC patients undergoing continued trans arterial chemoembolization (TACE) after TACE resistance.

Bernatz et al. [38] aimed to identify HCC patients who will respond to repetitive TACE to improve the treatment algorithm, extracting radiomics features from the 24 h post-embolization CT.

Zhang et al. [39] developed an interpretable machine learning model to predict the treatment response to initial conventional TACE (cTACE) in intermediate-stage HCC.

Wang et al. [40] established a transcriptomic biomarker for predicting the efficacy of TACE that correlates with radiomics features on pre-treatment imaging, tumor immune microenvironment characteristics, and the efficacy of immunotherapy and targeted therapy in HCC patients.

Recent studies investigated the role of radiomics in prediction of postoperative liver metastasis in pancreatic neuroendocrine tumor (panNET) after R0 resection [41].

Collectively, these studies underscore the potential of radiomics to inform pre-procedural decision-making in IR, while preclinical models provide a critical platform for biomarker validation and translational advancement.

Robust statistical or ML models are then developed and require independent validation to confirm their reliability and generalizability [15,26]. To guarantee methodological rigor, the quality of radiomics research should always be assessed using structured evaluation tools, such as the Radiomics Quality Score (RQS), which incorporates sixteen key components, or the more recent CheckList for EvaluAtion of Radiomics research (CLEAR) [15,26].

### 2.2. Virtual Reality in Learning

Virtual Reality, defined as a computer-generated immersive environment, has the potential to serve as a supplementary educational tool in medical training by enhancing the understanding of both preclinical and clinical concepts, as demonstrated by improvements in academic performance metrics [42].

VR offers significant advantages, including improved skill acquisition, cost-effectiveness, and the potential to reduce patient morbidity and mortality. Incorporating VR into medical education is anticipated to enhance theoretical understanding and foster the development of technical competencies essential for interventional radiology practice [42].

The spectrum of reality includes VR, mixed reality [43], and augmented reality (AR) and is reassumed in Table 3.

Virtual reality fully immerses people in an entirely virtual environment using headsets that cover the user’s entire field of vision. To interact with the environment, including virtual objects, one can use headsets, gloves, and earphones. Mixed reality consists in a hybrid approach that seamlessly blends virtual and real-world elements, allowing interaction between both in real time. MR uses advanced headsets to integrate and anchor digital objects in the real world, allowing dynamic interaction.

Augmented reality overlays digital content onto the real world, enhancing but not replacing the physical environment. AR uses devices like smartphones, tablets, or AR glasses to superimpose digital elements onto the real environment [44].

Simulations have already been adopted in orthopedic surgery training, typically using manual image segmentation and patient-specific anatomical models derived from cross-sectional imaging [2]. Similar VR-based simulation tools are being developed for IR training. Unique to IR is the need to cultivate tactile feedback, spatial reasoning, cognitive awareness, and motor skills essential for safe and effective equipment use [42]. In interventional radiology, VR can be used for training in angiography, angioplasty, vascular catheterization, catheter placement under fluoroscopic guidance, and stent placement and others [42].

The traditional apprenticeship model of “see one, do one, teach one” is increasingly being replaced by a “see many before doing many” approach, which reduces opportunities for direct procedural practice. Given the estimated 10,000 h required to attain expert-level proficiency [42,45], current training frameworks are often insufficient. VR simulation systems offer a potential solution by providing ample opportunities for deliberate practice within controlled environments.

Furthermore, variability in case mixes across institutions can lead to inconsistent training experiences. Simulation databases can expose trainees to a broader spectrum of cases, enhancing procedural preparedness. When combined with conventional teaching methods, VR systems can shorten procedure times, reduce operator error, and improve overall training outcomes in a safe and efficient manner [42,45].

Chaer et al. [46] conducted a randomized, controlled study with pre- and post-task questionnaires, with the aim to evaluate the acquisition of catheter skills by surgical residents with surgical simulation training comparing with specific instruction and didactic lectures, without simulations. The study revealed that simulator training improved the performance of residents in the operating room.

Knudsen et al. [47] designed a randomized, controlled, prospective study to validate the acquisition of percutaneous renal collecting system access skills using a computer-based hybrid virtual reality surgical (VRS) simulator compared to traditional resident training. The study revealed participants who received VRS training showed significant improvement in almost 80% of the parameters measured, whereas those in the control arm showed no significant improvement in any of the parameters [42].

VR also offers the potential to enhance IR training globally. In regions with limited access to specialized educators or training infrastructure, VR can provide high-quality, standardized education. Datasets used for training AI models could also serve as educational tools, complete with standardized reporting as answer keys. This approach would enrich educational resources and promote exposure to diverse anatomical and pathological cases, reducing the risk of bias in AI systems [8].

### 2.3. Advancing Medical Education Through Three-Dimensional (3D) Modeling

In IR, the incorporation of 3D modeling into AI learning procedures has become a game-changing strategy.

Using innovative biological tissue-mimicking resins, patient-specific vascular 3D printing makes it possible to reproduce intricate anatomy with extreme accuracy, producing lifelike physical models for pre-procedural planning and simulation.

By connecting imaging-derived quantitative features with clinical decision-making, these models improve predictive power in treatment response, recurrence, and survival outcomes, especially for liver cancers, when paired with AI-driven radiomics and texture analysis.

The learning process can offer not only pre-procedural planning and device testing but also potential ground-truth datasets for training AI algorithms to recognize vascular variations and predict treatment outcomes. These models can be combined with AI-driven radiomics and texture analysis, which extract quantitative features from imaging to enhance prognostic accuracy [48,49].

Furthermore, integrating 3D-printed models into advanced VR simulation platforms enables the creation of hybrid training environments, where AI systems adaptively tailor procedural difficulty and provide performance feedback. This synergy has already shown benefits in education, as VR-enhanced endovascular simulators significantly reduced fluoroscopy time and improved procedural proficiency among trainees. Collectively, 3D modeling strengthens the AI learning pipeline in IR by supplying anatomically precise data for algorithm development, validating predictive models, and fostering skill acquisition in a safe, iterative training environment.

Together, these advances highlight how 3D modeling not only strengthens the AI learning process by providing multimodal, data-rich environments but also bridges the gap between theoretical algorithms and practical, patient-centered interventions in IR [50].

The studies addressing pre-procedural applications of AI in interventional radiology, as discussed in this section, are summarized in Table 4, which provides an overview of the methodologies, imaging modalities, and key findings.

## 3. Intra-Procedural Applications of AI in IR

### 3.1. Cone-Beam Computed Tomography (CBCT) and Imaging Guidance Software

Cone-beam computed tomography has profoundly transformed interventional radiology by enabling high-resolution 3D imaging directly in the operating room [51,52]. When combined with image fusion technologies, CBCT facilitates multimodal visualization, significantly enhancing accuracy in both oncologic and vascular procedures [53,54].

Among the main advantages of CBCT is its capacity for intraoperative volumetric imaging. By providing real-time 3D reconstructions, CBCT improves target recognition and allows immediate post-procedural evaluation, as demonstrated in hepatic ablations or complex drainage procedures [51,55]. This capability is critical for verifying treatment completeness, such as tumor ablation or embolization [55,56,57].

**Figure 2:**
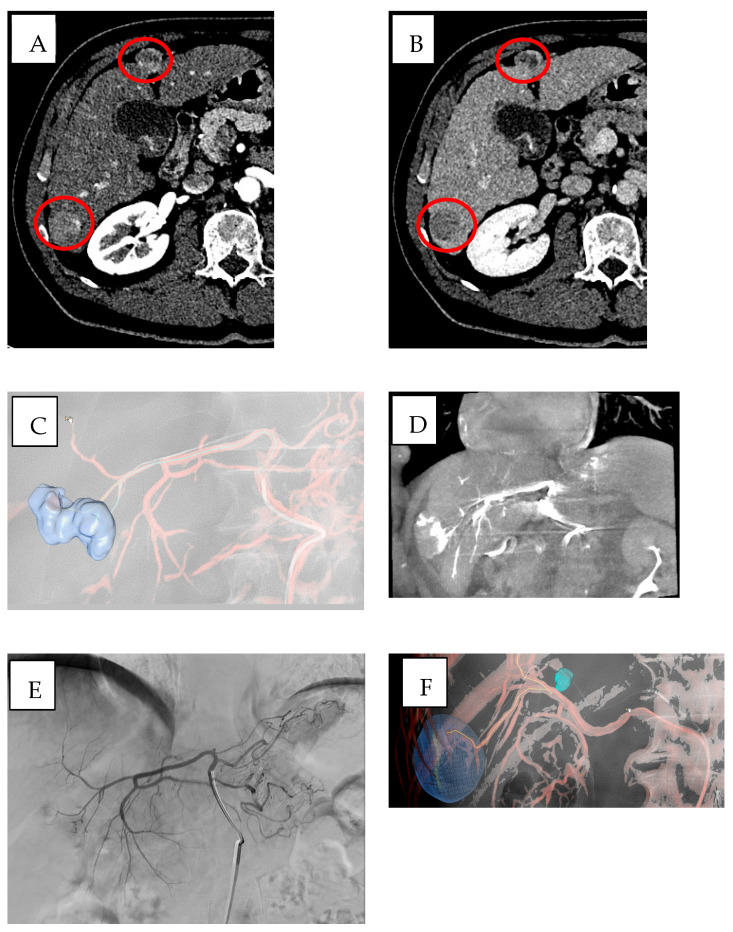

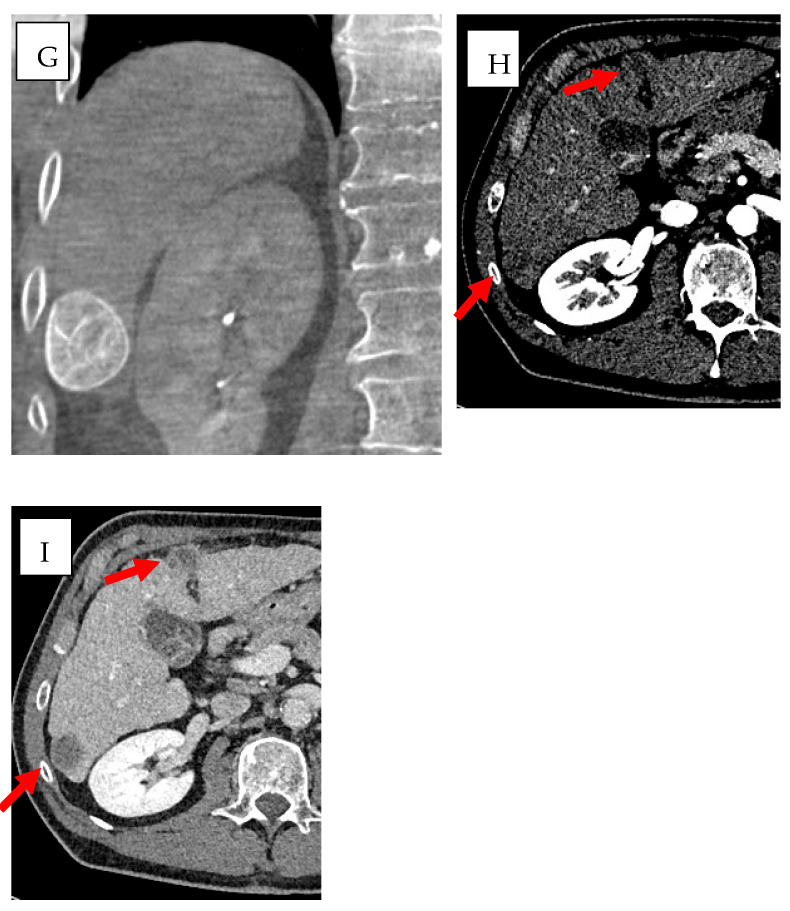
Multifocal HCC in a patient with HBV/HDV/alcohol-related cirrhosis (BCLC stage B) treated with DEB-TACE using BioPearl (Tokyo, Japan). (**A**,**B**) Pre-treatment CT shows two subcapsular lesions (~3 cm) in segments VI and IV with (**A**) arterial hyperenhancement and (**B**) venous washout (red circles), consistent with HCC. (**C**,**D**) Emboguide (Version 1.2.1, Philips) software and CBCT mapping of the arterial pathway from the right hepatic artery to the target lesion in segment IV. (**E**) Angiographic image showing the lesion in segment IV supplied by the left hepatic artery. (**F**,**G**) Emboguide (Philips) software and CBCT mapping of the right hepatic artery pathway to the lesion in segment VI before chemoembolization, showing enhancement of the target area. (**H**,**I**) Post-treatment CT in (**H**) arterial and (**I**) venous phases demonstrates hypodense areas (~3.5 cm) in segments VI and IV, consistent with treated lesions (red arrows).

In vascular procedures, CBCT can be used in addition to automated tumor feeder detection [58] to improve both safety and precision of embolization therapies. The AFD systems, achieving real-time 3D visualization of blood vessels through advanced imaging techniques with intelligent algorithms [59,60], consisting in three steps, in which the first step is the manual identification and segmentation of a ROI, the second is the manual identification of tip of catheter, and finally the last step is the automated identification of feeding arteries. The final 3D roadmap, containing the segmented ROI and feeding arteries and the paths from catheter to vessels, has been overlaid onto the live fluoroscopy images [61] (Figure 2).

These software utilize dynamic contrast-enhanced imaging for peak vessel visualization which enhances vascular structure definition, especially when dealing with tumors displaying irregular or abnormal blood supply. Real-time integration of these images enables precise catheter navigation which supports embolization procedures with reduced requirements for extensive fluoroscopy exposure [57,62].

In liver and lung tumor embolization, the AFD software enables accurate delivery of embolic agents to complex and variable tumor-feeding vessels, increasing treatment efficacy while minimizing non-target embolization and complications [59,60]. In neurovascular interventions, its ability to guide microcatheter navigation through intricate vascular networks supports safer and more effective treatment of aneurysms and arteriovenous malformations.

One of the key benefits is their ability to enhance the detection of small or tortuous feeder vessels, which are often difficult to identify using conventional digital subtraction angiography (DSA). The improved visualization and segmentation of the vascular anatomy—achieved through the use of contrast-enhanced CBCT—lead to more precise navigation and catheterization. This not only reduces the risk of missing target vessels but also limits the potential for non-target embolization [57,63,64].

Another significant advantage lies in the system’s capacity to shorten procedure times. By facilitating rapid and accurate feeder identification, AFD software reduces the need for repeated angiographic runs, minimizing radiation exposure for both the operator and the patient [65,66]. This improved workflow translates into greater procedural efficiency, particularly in high-volume interventional settings.

Despite these benefits, AFD software has some limitations. The accuracy of vessel segmentation and feeder identification is highly dependent on image quality. Suboptimal CBCT acquisitions—due to respiratory motion, patient obesity, or poor contrast opacification—can impair the system’s performance. Moreover, while the software provides semi-automated vessel mapping, operator input is often required to correct or refine suggested feeder paths, especially in complex anatomies [51].

Equally important is its precision in positioning. CBCT enables millimetric control of needle and catheter trajectories, minimizing the risk of technical errors or complications [55,64,67]. The technology also supports treatment verification, for example contrast-enhanced CBCT after tumor ablation of the liver allows early assessment of the ablation zone [68].

**Figure 3:**
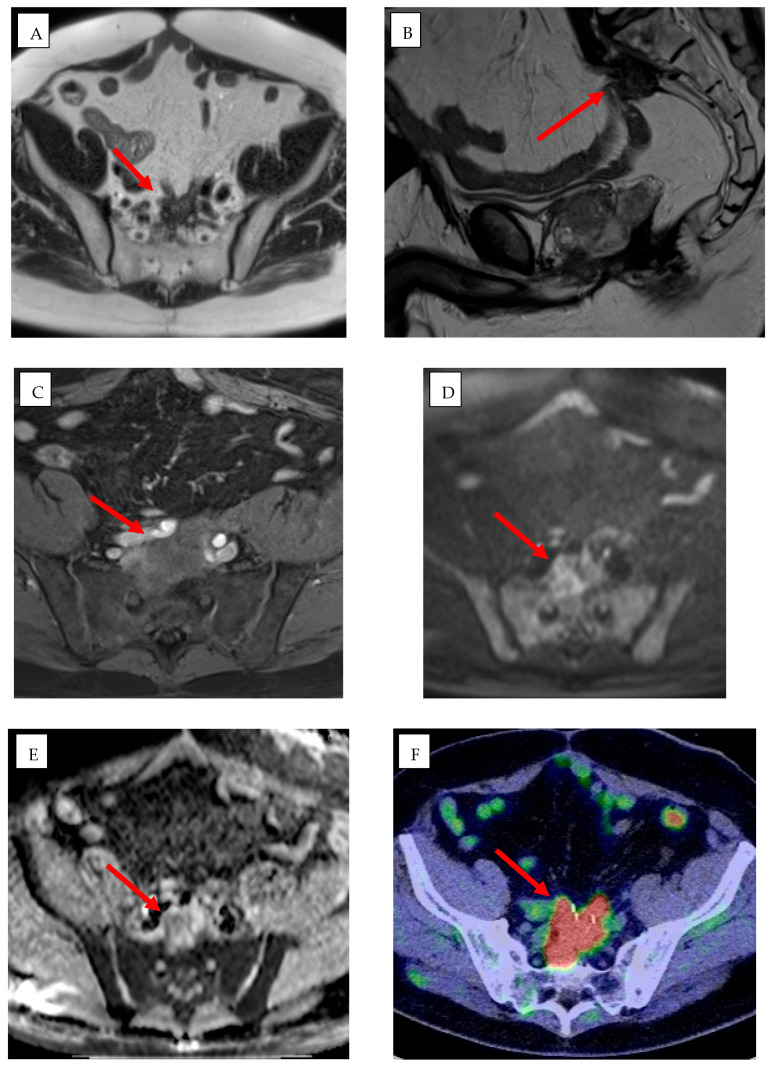

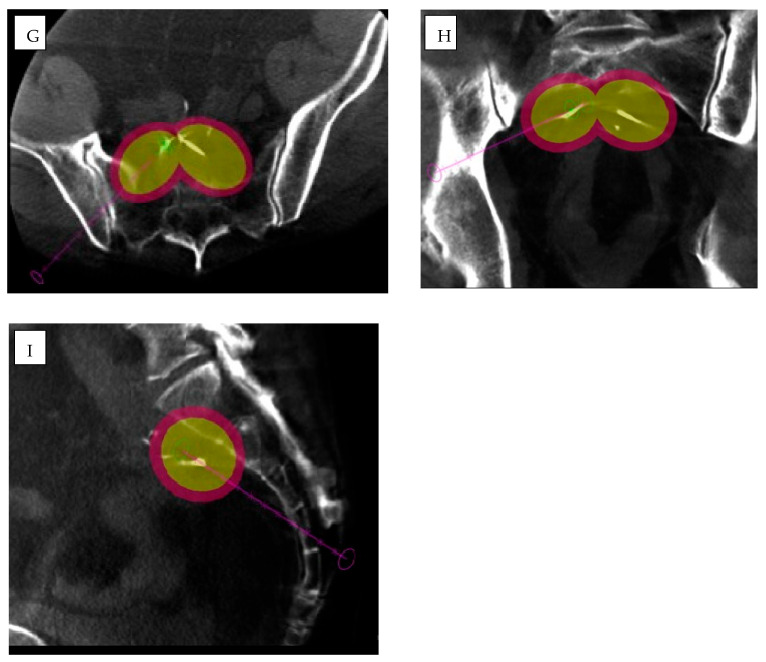
A 61-year-old man with rectal adenocarcinoma previously treated with surgery, adjuvant chemotherapy, and radiotherapy. Pre-procedural MRI, including axial (**A**) and sagittal (**B**) T2-weighted images, (**C**) T1-weighted post-contrast image, and (**D**) diffusion-weighted imaging (DWI) with (**E**) apparent diffusion coefficient (ADC) map, together with (**F**) 18F-FDG PET/CT, demonstrate a heterogeneous mass in the presacral region consistent with local disease recurrence (red arrows). (**G**–**I**) Intra-procedural cone-beam CT (CBCT) in axial (**G**), coronal (**H**), and sagittal (**I**) planes obtained using XperGuide software (Philips Healthcare) for cryoablation probe placement (purple arrows), and XperCT (Philips Healthcare) for ablation zone prediction (purple and yellow circles).

AI-driven image guidance systems such as XperGuide (Version 3.5.1, Philips Allura Xper FD20, Philips Healthcare), improve the precision of percutaneous procedures by delivering real-time three-dimensional needle guidance for percutaneous interventions [52,64] (Figure 3). XperCT software (Version 3.5.1, Philips Allura Xper FD20, Philips Healthcare) can be used to predict ablation volume [52].

XperGuide and XperCT are utilized in a wide variety of clinical settings, particularly in liver, lung, and renal tumor interventions. In liver tumors, particularly those located in the subphrenic region or near vital structures, their precise needle placement capabilities reduce the need for intraoperative patient repositioning and significantly lower the risk of complications such as infection and misplacement [53]. Similarly, in lung interventions, XperGuide enhances the accuracy of needle navigation in complex thoracic anatomies, thereby decreasing the number of passes required, reducing the risk of pneumothorax, and minimizing procedural trauma and subsequent interventions [64,69]. For renal tumors and spinal metastases, XperCT improves lesion visibility in areas where MRI may be limited, enabling more precise treatment planning and needle placement [64,70].

Both XperGuide and XperCT use intelligent algorithms for real-time trajectory optimization, motion compensation, and collision detection, but are not fully dependent on deep learning. These features enhance the safety of procedures by considering the patient movements—such as breathing—and avoiding important structures. Moreover, ongoing research seeks to integrate ML models that predict lesion response based on ablation geometry, suggesting optimal energy settings tailored to specific tissue characteristics. This would further personalize interventions, improving the likelihood of complete tumor ablation while minimizing damage to surrounding healthy tissues [51,54].

### 3.2. Intraoperative Applications of VR

The intraoperative applications of VR are numerous and increasingly sophisticated. One of the most promising is real-time navigation and guidance, where VR is integrated with live imaging modalities such as fluoroscopy, ultrasound, or CBCT. This fusion enables dynamic navigation during a procedure, improving spatial orientation and precision, and allowing procedural strategies to be adapted instantly to patient-specific anatomical variations.

Closely related is the use of AR overlays, where hybrid AR–VR solutions project virtual elements directly onto the sterile field. This approach improves ergonomics and safety during critical steps, such as needle placement in percutaneous ablations, while reducing the cognitive load on clinicians by simplifying complex decision-making processes [71].

Another frontier is the integration of AI into VR systems. AI algorithms can automatically evaluate anatomical variants, suggest optimal trajectories for vascular access or needle insertion, and provide real-time predictive analytics to anticipate potential complications or deviations from the ideal path [8].

VR also facilitates remote collaboration. By creating shared virtual environments, multidisciplinary teams can interact in real time from different locations, supporting complex interventions and enabling expert consultation even in remote or resource-limited settings [72].

Some platforms now offer dynamic personalized simulation, where intraoperative images continuously update the virtual model, allowing real-time adjustments to the planned strategy and enabling a more adaptive, patient-specific approach to treatment [73,74].

Advanced anatomical visualization represents another strength of VR. Through stereoscopic interaction with reconstructed organs and vessels, operators achieve more precise targeting in procedures such as TACE, radioembolization, and tumor ablations. This three-dimensional immersion enhances depth perception and targeting accuracy compared to traditional 2D imaging [72].

A particularly innovative intraoperative use is procedural rehearsal, where clinicians can simulate key procedural steps on a patient’s anatomical model shortly before execution. This allows them to anticipate potential challenges and fine-tune their approach in real time, improving overall procedural efficiency [75].

Similar to hypnosis, which has already proven its effectiveness in analgesic treatment as a distraction technique, VR has recently emerged as a new therapeutic weapon, also during IR procedures. Grange et al. [76] evaluated the tolerance and feasibility of using VR headsets with patients during interventional radiology procedures, demonstrating that it can be beneficial for pain and anxiety management.

The introduction of VR into clinical practice also raises ethical and practical considerations. Patient privacy and data security are paramount, as VR platforms rely on highly sensitive, patient-specific data. Compliance with regulations such as the GDPR in Europe and HIPAA in the United States is mandatory [77], and patients must be fully informed about how their data will be used, particularly when VR is employed for training or planning [78]. Ensuring adequate clinician training is also critical to avoid misuse or over-reliance on the technology, which could lead to errors. While VR holds great promise for improving procedural precision and outcomes, the long-term impact of VR-based training on real-world clinical performance remains to be fully evaluated. There are also potential psychological considerations, such as disorientation or dependency on immersive environments [79].

Despite its potential, VR adoption faces technical and practical challenges. Rendering latency, headset ergonomics, compatibility with existing medical infrastructure, and high costs still limit widespread use [42]. Additionally, specialized staff training is essential, and concerns persist regarding data storage, privacy, and regulatory compliance. Nevertheless, with ongoing technological advances and increasing clinical demand, costs are expected to decrease, facilitating broader clinical integration.

### 3.3. Robotics

Various robotic devices (table-mounted, floor-mounted, gantry-mounted, and patient-mounted) have been developed to enhance precision and standardization in procedures. These systems can provide real-time visualization and tracking, allowing for better trajectory planning and needle placement; robotic systems have evolved to allow for multiple degrees of freedom, enhancing their flexibility and precision.

- Table-Mounted Systems: These systems manipulate the needle under imaging guidance and have shown high accuracy in clinical settings [80];
- Floor-Mounted Systems: These devices can hold and orient needles and have demonstrated improved accuracy in phantom and animal studies [80];
- Patient-Mounted Systems: These systems offer ergonomic advantages and have shown promising results in clinical trials [80].

Interventional radiology robots can support both percutaneous and endovascular procedures under various imaging modalities, including CT, MRI, US, and fluoroscopy, as well as through fused multimodal image datasets [61,81,82].

#### 3.3.1. Percutaneous Applications

Accurate visualization of the target via CT, CBCT, MRI, fluoroscopy, and US is critical for percutaneous procedures—biopsy, tumor ablation, and infiltration.

CT/CBCT guidance enables precise image-guided interventions, while navigation software and robotic assistance further enhance targeting accuracy, shorten procedure time, and lower radiation exposure. Commercial platforms such as Maxio and iSYS report improved needle-insertion times and sub-2 mm targeting errors; XACT Robotics demonstrated <8.5 min skin-to-target times and robust compensation for respiratory motion [83]. MRI-compatible robots leverage high soft-tissue contrast and eliminate ionizing radiation, although spatial constraints and material compatibility remain challenges. US-guided systems (e.g., B-Rob I) automate probe stabilization and needle placement to overcome operator dependence [84,85].

Although less established than CT, needle-based techniques can be integrated with MRI to accurately locate anatomical structures. MRI-guided robotic systems are primarily utilized for biopsies and ablations in prostate, brain, and breast cancer cases. A drawback of this approach is the challenge of positioning and maneuvering the needle within a closed-bore MRI scanner, necessitating that the patient be moved in and out of the scanner for these procedures. Additionally, transrectal MRI-guided prostate biopsies may offer a quicker alternative compared to manually adjusting the needle; robotic-assisted MRI-guided biopsy yields 100% technical success rate with a short MRI room occupation time [10,86].

Zheng et al. demonstrated excellent results in assisted percutaneous discectomy with the Mazor X robotic system that utilizes three-dimensional CT imaging for surgical trajectory design, allowing precise planning of access angles and diameters for the procedure. Under fluoroscopic guide visualization, the system guides the puncture dilation and instrument insertion along the defined surgical path [87].

#### 3.3.2. Endovascular Applications

Endovascular robotic systems, first introduced in the mid-2000s, were developed to enhance catheter stability, improve procedural precision, and reduce radiation exposure for the clinician [88]. Early platforms—such as the Sensei X (Hansen Medical), Niobe (Stereotaxis), and Amigo RCS (Catheter Robotics)—demonstrated feasibility for robot-assisted navigation of large-bore catheters in cardiac and aortic procedures. However, these systems were limited by their bulky design, lack of tactile feedback, and prolonged setup times, making them unsuitable for navigating smaller, tortuous vessels or for use in urgent clinical scenarios [89,90,91].

Second-generation systems, such as the Magellan (Hansen) and CorPath 200/GRX (Corindus), have since introduced slimmer, more flexible robotic catheters offering multiple degrees of freedom, thereby expanding the scope of endovascular applications [61]. These platforms have been successfully applied in procedures such as uterine artery embolization, hepatic chemoembolization, and peripheral revascularization, demonstrating high technical success and significant reductions in radiation exposure for operators [92].

Despite these advances, key limitations persist. Most systems still lack true haptic or force feedback, depend on 2D fluoroscopic guidance, and perform suboptimally in thrombosed or highly tortuous vasculature. Current research is therefore shifting toward next-generation technologies, including untethered microrobots and soft-body actuators—self-propelled, wireless devices capable of autonomous navigation—which may enable fully minimally invasive, radiation-free interventions in the future [93].

Robotic platforms also show promise as training tools when integrated with surgical simulators, offering realistic procedural practice while minimizing radiation exposure. Additionally, robotic assistance may help reduce inter-operator variability. However, widespread clinical adoption remains hindered by high system costs, limited compatibility with existing tools, workflow disruption in interventional suites, and the continued absence of haptic feedback [94].

### 3.4. Imaging Fusion

**Figure 4:**
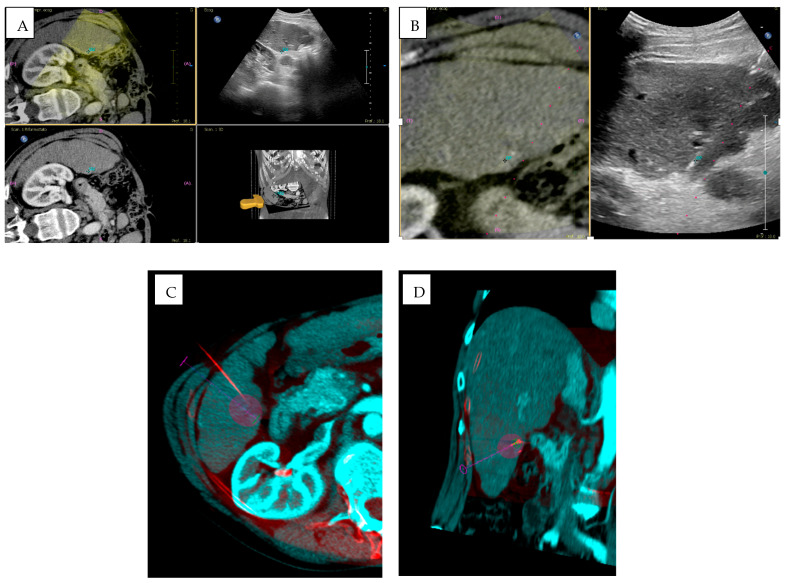
A 72-year-old man with HCC treated with MWTA under fusion imaging guidance. (**A**) Pre-procedural planning using fusion of CT and ultrasound images to identify the target lesion. (**B**) Intra-procedural monitoring during insertion of the MWTA antenna into the target lesion. (**C**,**D**) Prediction of the ablation zone using XperCT software (EPIQ PercuNav, Philips fusion system) (purple circles).

**Figure 5:**
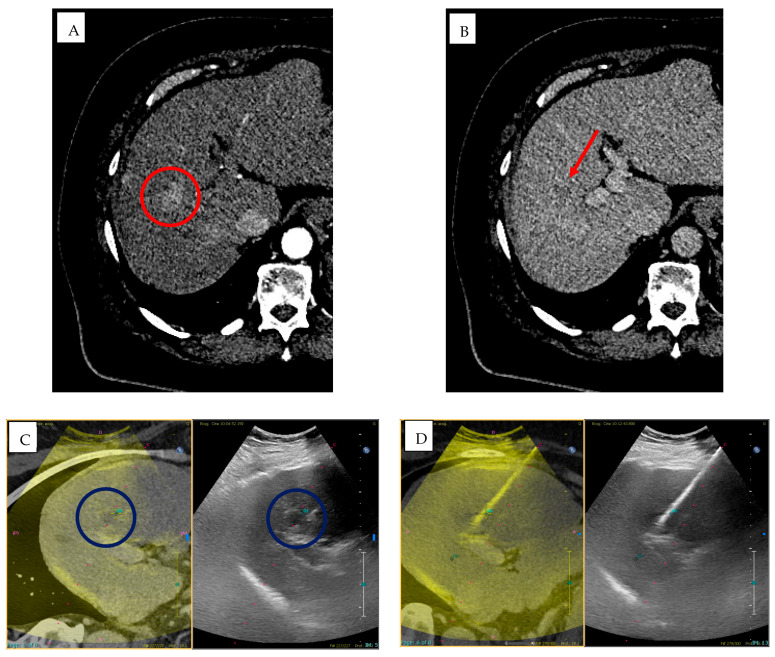

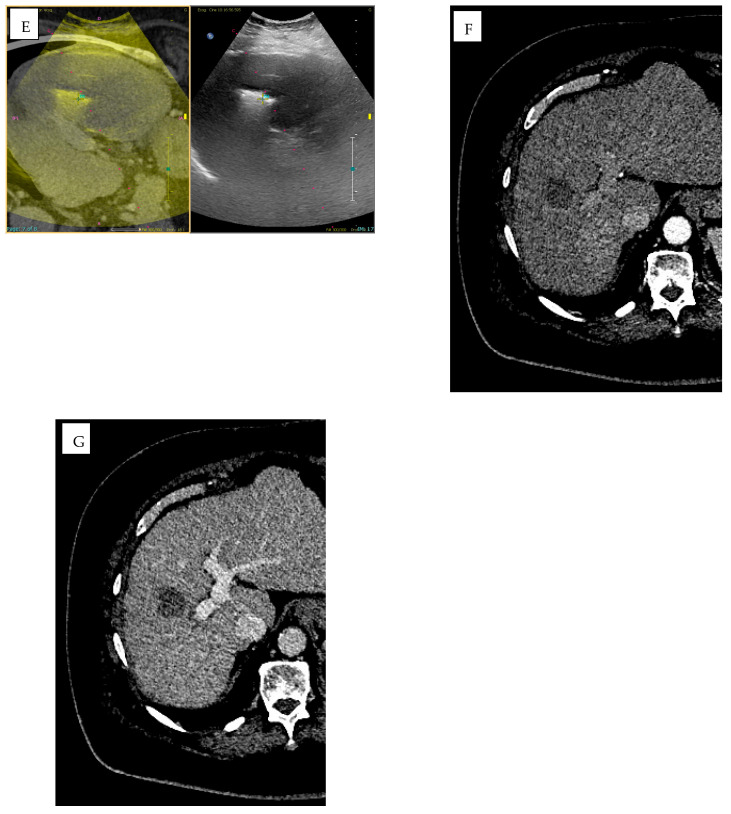
A 71-year-old man with HCC in the setting of NASH-related liver disease, previously treated with MWTA under fusion imaging guidance. (**A**,**B**) Pre-treatment CT images show a 2 cm lesion in segment VII of the liver, demonstrating arterial phase hyperenhancement (red circle) (**A**) and venous phase washout (red arrow) (**B**). (**C**) Fusion imaging (EPIQ PercuNav, Philips) showing selection of the target lesion (blue circles) in segment VII using ultrasound–CT co-registration. (**D**) Introduction of the MWTA antenna (20 cm length) into the target lesion; ablation performed for 2 min 30 s at 150 W. (**E**) Intra-procedural monitoring demonstrates the typical “popcorn effect” of the hepatic parenchyma caused by tissue heating and vaporization. (**F**,**G**) Post-treatment CT images in the arterial (**F**) and venous (**G**) phases demonstrate a 3 cm hypodense area in segment VII, consistent with complete response at 1-month follow-up.

Fusion imaging is a technique based on the integration of different imaging modalities, with the aim to enhance the power of each one, reducing to a minimum the weaknesses of each individual mode. The process consists of different steps. The first step is importation of data from a previous CT/MR/PET exam, followed by the spatial alignment of the imaging dataset and both anatomical landmarks and external markers can be used. Imaging registration can be carried manually by the operator, automatically based on matching common anatomical landmarks, or semi-automatically using a combination of both techniques. When an appropriate alignment is achieved, real-time US and CT/MR/PET images are overlaid on the US monitor, displaying the same plane and moving synchronously together [95] (Figure 4 and Figure 5).

A major enhancement to CBCT is its integration with image fusion. The alignment between preoperative CT and intraoperative CBCT improves visualization of hepatic lesions and facilitates accurate hepatic segmentation [96]. Multimodal fusion-guided navigation merges preoperative images with fluoroscopy or CBCT for advanced 3D orientation, aiding in selective chemoembolization, complex biopsies, and vascular malformation treatments [54,97,98]. By improving targeting, fusion technologies can also reduce procedural time, lowering radiation exposure for both patients and operators [99].

Modern systems now offer automatic, dynamic registration, aligning pre- and intraoperative datasets without manual intervention and ensuring smooth transitions between modalities [98]. The clinical applications of CBCT and image fusion are extensive. In liver oncology, this combination is becoming the emerging standard for TACE and ablation procedures, allowing precise targeting of lesions that are difficult to visualize with a single modality [96,100,101]. In vascular interventions, CBCT with vascular overlay improves embolization planning and stent placement in complex anatomies [101]. Nonetheless, there are limitations and considerations. CBCT is susceptible to motion artifacts, particularly in non-cooperative patients, and involves higher radiation doses than ultrasound—though lower than conventional CT [67]. Image fusion accuracy heavily depends on precise registration; even small errors can affect targeting. Finally, proper use of CBCT and fusion systems requires specialized training and significant financial investment, which may be challenging for smaller healthcare facilities.

The main studies exploring intra-procedural applications of AI, including CBCT, VR, robotics, and fusion imaging are summarized in Table 5, highlighting their methodological approaches and clinical impact.

## 4. Post-Procedural Applications of AI in IR

Artificial intelligence methodologies, encompassing machine learning and deep learning, are increasingly utilized in the analysis of post-procedural imaging to assess treatment efficacy and predict clinical outcomes. These technologies contribute to enhanced workflow efficiency, reduced inter-observer variability in image interpretation, and improved accuracy in post-procedural assessment. Moreover, AI plays a pivotal role in the quantification of treatment response, supports prognostic evaluation, and informs subsequent management strategies [102]. Abajian et al. demonstrated the use of random forest models incorporating MRI signal intensity, contrast enhancement, and clinical parameters (e.g., cirrhosis) to classify responses following TACE, achieving promising predictive performance [3].

Moon et al. [24] evaluated the potential of four-dimensional (4D) flow MRI in predicting treatment responses after TACE in cirrhotic patients with HCC, revealing that the quantitative flow data obtained by 4D flow MRI may be useful for predicting CR after TACE in cirrhotic patients with HCC.

Similarly, support vector machine models based on radiomic features from post-EVAR CT angiography effectively identified aggressive type II endoleaks associated with aneurysmal sac expansion, reinforcing the role of AI in personalized surveillance strategies [103].

In a pilot study, Daye et al. integrated clinical and radiomic data from pre-treatment CT scans to predict local tumor progression and survival in patients undergoing percutaneous thermal ablation for adrenal metastases, demonstrating high predictive accuracy [104]. Additional work by Sinha et al. showed the potential of machine learning to forecast procedure-specific outcomes such as pneumothorax after CT-guided transthoracic biopsy, in-hospital mortality post-TIPS, and prolonged hospital stay following uterine artery embolization [105].

Machine learning algorithms have also been used to predict long-term complications after inferior vena cava (IVC) filter placement by integrating a wide array of clinical, anatomical, and device-related variables. These models may enhance patient selection, perioperative planning, and post-procedural follow-up, ultimately reducing complication rates [43].

Beyond predictive modeling, AI holds potential to streamline post-treatment image analysis and improve inter-observer consistency. The generation of quantitative imaging biomarkers supports tailored treatment strategies and facilitates more nuanced risk stratification. In clinical practice, interventional radiologists frequently engage in multidisciplinary discussions to integrate AI-derived insights with comprehensive patient data, optimizing both immediate therapeutic decisions and long-term follow-up plans [1].

For clarity, the studies described in this section are summarized in Table 6, which provides a structured overview of their methodologies, imaging modalities, and principal contributions to AI in IR.

## 5. Discussion

The integration of AI into IR is progressing rapidly and has the potential to remake the field by upgrading efficiency, accuracy, and personalization of treatments. AI can improve decision-making, procedural guidance, and long-term patient management with different tools used during pre-, intra-, and post-procedures.

Artificial intelligence has been firstly adopted for diagnostic radiology, mainly with application in automated detection of findings and features, automated interpretation of findings, and post-processing imaging tools such as reduction in image noise and artifacts [106,107].

Artificial intelligence tools for diagnostic radiology are supported by large, annotated datasets and standardized imaging protocols. Several studies have assessed the role of radiomics in the extraction of features to predict a response to a specific treatment [108,109,110,111,112,113].

In contrast, AI adoption in IR is still at the beginning. The interventional environment presents considerable complexity due to variability in procedures, different techniques and devices, and real-time nature of intraoperative imaging. In fact, unlike diagnostic radiology, based on static images, IR requires continuous integration of multimodal data—cross-sectional imaging, fluoroscopy, ultrasound, and CBCT. This complexity renders the development of algorithms specific for IR settings significantly more challenging.

Artificial intelligence in IR can gain advances from diagnostic radiology. Radiomics, already used in diagnostic imaging for tumor characterization and prognosis, can be applied to procedural planning in in patient selection for IR procedure in order to personalize therapies [114,115,116]. Workflow optimization algorithms, widely used in diagnostic radiology for planning and reporting, could be tailored to facilitate procedure scheduling, reduce delays, and improve resource allocation in IR [117].

The intraoperative environment of IR provides different opportunities to validate the usefulness of AI. Image fusion and CBCT navigation systems, could reduce procedure times, radiation dose, enhancing the efficacy of the procedure. Robotics, supported by machine learning, can improve precision and reproducibility of procedures. After the procedure, predictive models can aid in monitoring outcomes, anticipating complications, and guiding follow-up strategies, parallel to predictive analytics already proven in diagnostic radiology [118,119,120].

Virtual reality, combined with 3D modelling, is revolutionizing medical education and simulation-based training, surpassing the limitations of traditional educational models [74].

However, several barriers still hinder the full implementation of AI in daily interventional radiology practice. Data heterogeneity remains a major limitation, as inter-institutional differences in imaging protocols, device selection, and procedural techniques make it challenging to develop generalizable algorithms. Moreover, most studies in IR are constrained by small sample sizes and retrospective designs, limiting the robustness and reproducibility of current evidence. AI integration is nevertheless expected to gradually overcome these challenges through multicenter data sharing, federated learning, and harmonization algorithms capable of standardizing imaging acquisition and analysis across platforms. As automation increases and technology matures, implementation costs are anticipated to decline, facilitating broader clinical adoption [121,122]. In addition, the current level of algorithmic sophistication may not yet allow AI systems to reliably identify the optimal procedural approach, particularly in complex or variable interventional settings. In this regard, it may be more appropriate in the near future to refer to the concept of “hybrid intelligence,” in which AI supports and accelerates specific procedural steps, while human expertise remains essential for final decision-making. Robust multicenter studies and standardized datasets are required to determine clinical utility and reproducibility of AI software [15]. Ethical and legal concerns further complicate the implementation of AI in medicine, particularly regarding data privacy, accountability in cases of diagnostic or therapeutic errors, and the need for transparent decision-making processes [58,123,124]. The ongoing debate centers on how AI will transform clinical roles and responsibilities, raising crucial questions of liability when AI-assisted decisions lead to adverse outcomes. Current legal frameworks hold supervising physicians strictly liable, emphasizing the necessity for clear regulations defining accountability in AI-supported practice. To minimize errors, AI algorithms and their underlying datasets should undergo regular validation and updates, while patients must be adequately informed about the use of AI systems in their care [123]. Together, these challenges highlight the need for more standardized data collection, rigorous clinical research, and clear regulatory frameworks before AI can be fully integrated into interventional radiology practice.

## 6. Future Perspectives

The integration of AI into IR offers transformative potential while raising important ethical challenges related to data governance, algorithm and model development, and clinical practice. Establishing standardized research and implementation practices is essential to ensure consistency, transparency, and reliability in AI-driven applications. As virtual healthcare expands, patient–clinician interactions have intensified, increasing professional workload; AI-based assistants could help address this demand by generating structured responses to patient queries and contributing to patient education and management. In parallel, the combination of robotics and AI promises to enhance the precision, efficiency, and clinical outcomes of IR procedures. Furthermore, AI-driven innovations are transforming clinical research by enabling the generation of high-quality synthetic datasets, thereby accelerating trial design and fostering faster translation of novel therapies. Looking ahead, the establishment of international networks and collaborative task forces will be essential in disseminating expertise, supporting institutions in building capabilities, and ensuring that the benefits of AI are equitably shared across the global IR community.

## 7. Conclusions

Artificial intelligence is rapidly transforming the landscape of IR by enhancing precision, efficiency, and personalization across pre-, intra-, and post-procedural phases. Radiomics enables noninvasive extraction of imaging biomarkers that can inform patient selection and optimize treatment strategies. Virtual and augmented reality, combined with 3D modeling, are reshaping medical education and procedural simulation, providing safe and reproducible training environments. Intra-procedural applications, including CBCT, image fusion, and robotic-assisted systems, demonstrate the potential to improve navigation accuracy, reduce radiation exposure, and increase procedural reproducibility. Post-procedural AI tools support objective treatment assessment, predictive outcome modeling, and personalized follow-up strategies.

Despite this promise, widespread clinical adoption of AI in interventional radiology remains limited by data heterogeneity, small study populations, and ethical and regulatory challenges. Multicenter collaborations, standardized datasets, and rigorous prospective trials will be essential to validate existing applications and ensure reproducibility. Furthermore, clear legal frameworks and transparency in algorithmic decision-making are required to address patient safety and accountability.

Looking ahead, the integration of AI with robotics, extended reality, and advanced imaging modalities has the potential to redefine the practice of interventional radiology. By bridging technical innovation with clinical needs, AI may ultimately contribute to safer procedures, more consistent outcomes, and a higher degree of personalization in patient care.

## Figures and Tables

**Table 1:** Summary of pre-procedural AI applications in interventional radiology.

Application Area	AI/Technology Used	Purpose	Key Outcomes/Findings
**Radiomics**	ML-based image analysis	Prediction and patient stratification	Improved diagnosis, prognosis, therapy planning; validated biomarkers
**VR**	Immersive simulation	Skill development and training	Enhanced proficiency, reduced errors, global accessibility
**3D Modeling**	3D printing + AI integration	Preprocedural planning, algorithm training	Realistic anatomy models, better predictive accuracy, hybrid learning environments

**Table 2:** Summary of intra-procedural AI applications in interventional radiology.

Category/ Technology	Main Applications	AI Functions/ Automation	Clinical Advantages	Main Limitations
**IMAGING GUIDANCE SOFTWARES**				
Cone-Beam CT (CBCT) and Automated Feeder Detection (AFD) Systems	Endovascular procedures	Real-time 3D reconstruction, automated tumor feeder detection, vessel segmentation	Improved target vessel recognition and treatment verification; enhanced embolization precision; reduced radiation dose and procedure time	Sensitive to image quality (motion, contrast); requires manual input for complex anatomy
XperGuide/XperCT (Philips)	Percutaneous iprocedures	Real-time needle trajectory optimization, ablation volume prediction	Higher accuracy of needle placement; reduced complications, repositioning,	Not fully deep learning-based; high cost
Virtual Reality (VR)/Augmented Reality (AR) Systems	Intraoperative navigation, real-time guidance, training, remote collaboration	AI-enhanced spatial mapping, trajectory suggestion, complication prediction	Improved spatial orientation and depth perception; dynamic adaptation to anatomy; analgesic and anxiety reduction	High cost, rendering latency, need for operator training, data privacy concerns
**ROBOTICS**				
Percutaneous Robotic Systems (e.g., Maxio, iSYS, XACT, Mazor X)	Biopsy, ablation, infiltration, discectomy	Automated trajectory planning, robotic needle insertion, respiratory motion compensation	Sub-2 mm targeting accuracy; shorter procedure time; reduced operator radiation exposure	MRI compatibility limitations; lack of tactile feedback; high system cost
Endovascular Robotic Systems (e.g., Magellan, CorPath, Sensei X)	Embolization, revascularization, cardiac and aortic interventions	AI-assisted catheter navigation and path optimization	Enhanced catheter stability and precision; reduced operator radiation dose	Lack of haptic feedback; long setup time; limited flexibility in tortuous anatomy
**IMAGING FUSION**				
Multimodal Fusion Imaging (CT/MR/PET + US/CBCT)	TACE, ablation, biopsies, vascular malformations	AI-supported registration and alignment of multimodal datasets; real-time anatomical matching	Improved lesion localization and targeting; reduced procedural time and radiation exposure	Accuracy depends on registration precision; requires training and expensive equipment

**Table 3:** The table summarizes the characteristics of the spectrum of reality including virtual reality (VR), mixed reality (MR) and augmented reality (AR).

Type	Definition	Key Features/Functions	Devices Used
VR	Full immersion in a completely virtual environment.	-Replaces the real world entirely with a simulated one. -Users can interact with virtual objects.	Headsets covering the entire field of vision, gloves, earphones.
MR	Hybrid approach combining real and virtual elements.	-Real-time interaction between real and digital worlds. -Digital objects are integrated and anchored in the real environment.	Advanced headsets enabling seamless blending and interaction.
AR	Overlays digital content onto the real world.	-Enhances but does not replace the physical environment. -Superimposes digital elements on real surroundings.	Smartphones, tablets, AR glasses.

**Table 4:** Pre-procedural applications of AI in IR.

Application Category	Author	Methodology	Imaging Modality	Key Findings
Radiomics	Mamone et al. [33]	Radiomics	Pre-procedural CT	Predicted survival, hepatic encephalopathy, and clinical response after TIPS creation
	Tabari et al. [34]	Radiomics + ML	Pre-ablation MRI	Predicted pathological response in HCC patients undergoing transplantation
	Li et al. [35]	Radiomics	MRI/CT	Demonstrated role of radiomics in forecasting microvascular invasion (MVI) in HCC
	He et al. [36]	Prognostic nomogram integrating radiomics	Pre-procedural CT (HCC after surgery)	Evaluated survival benefit of HCC patients receiving adjuvant TACE
	Yang et al. [37]	Radiomics-based survival model	Pre-treatment enhanced MRI	Predicted prognosis of HCC patients undergoing continued TACE after resistance
	Bernatz et al. [38]	Radiomics feature extraction + ML	Post-embolization CT	Identified HCC patients responding to repetitive TACE
	Zhang et al. [39]	Interpretable ML model	Contrast-enhanced CT	Predicted treatment response to initial cTACE in intermediate-stage HCC
	Wang et al. [40]	Radiogenomic approach (radiomics + transcriptomics)	Pre-treatment imaging	Transcriptomic biomarker correlated with radiomics features to predict TACE efficacy and immunotherapy outcomes
	Ma et al. [41]	Radiomics + DL	Pre-op CT	Predicted postoperative liver metastasis in pancreatic neuroendocrine tumor (panNET) after R0 resection
	Mosconi et al. [13]	Radiomics + ML	Pre-treatment CT	Predicted responders to radioembolization in cholangiocarcinoma
	Avery et al. [26]	Radiomics Quality Score (RQS)	N/A	Provided 16-component framework for evaluating radiomics research
	Ferrari et al. [15]	CLEAR checklist	N/A	Introduced structured criteria to ensure methodological rigor and reproducibility in radiomics studies
Virtual Reality	Waller et al. [2]	Review of AI applications	N/A	Overview of AI opportunities and challenges in diagnostic and interventional radiology
	Gelmini et al. [42]	Systematic review (VR in IR training)	VR-based simulation VR-based simulation	Demonstrated improved skill acquisition, cost-effectiveness, and reduced morbidity/mortality risk
	Li et al. [43]	ML predictive models	CT/clinical data	Used ML to predict IVC filter complications; findings relevant to pre-procedural planning
	Tortora et al. [44]	Extended reality (AR/VR/MR)	Simulation and imaging	Reviewed applications of extended reality in radiology and discussed future perspectives
	Chaer et al. [46]	VR simulation training	Simulation (angiography, catheter skills)	Simulator training improved surgical residents’ performance compared with didactic instruction
	Knudsen et al. [47]	Hybrid VR simulator	Computer-based surgical simulation	Improved acquisition of percutaneous renal access skills compared with traditional training
3D Modeling	Kaufmann et al. [48]	3D vascular printing with AI integration	CT/MRI-derived 3D models	Patient-specific anatomical models improved pre-procedural planning and provided datasets for AI training
	Tenewitz et al. [49]	Systematic review of 3D modeling in IR training	3D printed vascular models	Demonstrated feasibility and educational value of 3D printing for IR trainees

**Table 5:** Intra-procedural applications of AI in IR.

Application Category	Subcategory	Author	Methodology	Imaging Modality	Clinical Application	Key Findings
CBCT and AFD	Imaging guidance/feeder detection	Abi-Jaoudeh et al. [97]	AFD (feeder detection) and vessel segmentation	CBCT, CT, US, Fluoroscopy	Embolization (TACE/TAE), vascular malformation	Improves targeting and procedural accuracy.
	Imaging guidance/feeder detection	Schernthaner et al. [66]	AFD (feeder detection) and vessel segmentation	CBCT/Fluoroscopy	Ablation/embolization (general)	Shortens procedures and reduces radiation.
	Imaging guidance/feeder detection	Wallace et al. [70]	AFD (feeder detection) and vessel segmentation	CT, MRI	Renal tumor	Improves targeting and procedural accuracy.
	Imaging guidance/feeder detection	Chiaradia et al. [59]	AFD (feeder detection) and vessel segmentation	CBCT/Fluoroscopy	Ablation/embolization (general)	Enhances feeder detection and embolization planning.
	Imaging guidance/feeder detection	Barral et al. [51]	AFD (feeder detection) and vessel segmentation	CT	Ablation/embolization (general)	Enhances feeder detection and embolization planning.
	Imaging guidance/feeder detection	Monfardini et al. [53]	AFD (feeder detection) and vessel segmentation	CBCT, CT, US	Ablation/embolization (general)	Improves targeting and procedural accuracy.
	Imaging guidance/feeder detection	Shinde et al. [65]	AFD (feeder detection) and vessel segmentation	CBCT/Fluoroscopy	Ablation/embolization (general)	Shortens procedures and reduces radiation.
	Imaging guidance/feeder detection	Lanza et al. [61]	AFD (feeder detection) and vessel segmentation	Fluoroscopy	Ablation/embolization (general)	Improves targeting and procedural accuracy.
	Imaging guidance/feeder detection	Abdelsalam et al. [60]	AFD (feeder detection) and vessel segmentation	CBCT/Fluoroscopy	Ablation/embolization (general)	Enhances feeder detection and embolization planning.
	Imaging guidance/feeder detection	Busser et al. [69]	AFD (feeder detection) and vessel segmentation	CBCT/Fluoroscopy	Lung tumor	Shortens procedures and reduces radiation. Improves targeting and procedural accuracy.
	Imaging guidance/feeder detection	Racadio et al. [52]	AFD (feeder detection) and vessel segmentation	CT	Ablation/embolization (general)	Enhances feeder detection and embolization planning.
	Imaging guidance/feeder detection	Braak et al. [64]	AFD (feeder detection) and vessel segmentation	CBCT/Fluoroscopy	Embolization (TACE/TAE)	Shortens procedures and reduces radiation. Improves targeting and procedural accuracy.
	Imaging guidance/feeder detection	Key et al. [54]	AFD (feeder detection) and vessel segmentation	CBCT, CT, US	Ablation/embolization (general)	Improves targeting and procedural accuracy.
	Imaging guidance/feeder detection	Zeiler et al. [63]	AFD (feeder detection) and vessel segmentation	CBCT/Fluoroscopy	Embolization (TACE/TAE)	Shortens procedures and reduces radiation. Improves targeting and procedural accuracy.
	Imaging guidance/feeder detection	Kim et al. [62]	AFD (feeder detection) and vessel segmentation	Fluoroscopy	Embolization (TACE/TAE)	Shortens procedures and reduces radiation. Improves targeting and procedural accuracy.
	Imaging guidance/feeder detection	Tacher et al. [98]	AFD (feeder detection) and vessel segmentation	CBCT, CT, US, Fluoroscopy	Embolization (TACE/TAE), vascular malformation	Improves targeting and procedural accuracy.
	Treatment verification	Tacher et al. [57]	AFD (feeder detection) and vessel segmentation	CBCT/Fluoroscopy	Ablation, embolization (TACE/TAE)	Enhances feeder detection and embolization planning.
	Treatment verification	Morimoto et al. [55]	AFD (feeder detection) and vessel segmentation	CBCT, CT	Ablation	Improves targeting and procedural accuracy.
Fusion Imaging	Clinical fusion applications	Angle et al. [101]	Automatic/semiautomatic registration for multimodal fusion	CT	Ablation, liver tumor	Improves targeting and procedural accuracy.
	Clinical fusion applications	Zhong et al. [100]	Automatic/semiautomatic registration for multimodal fusion	CT	Ablation, liver tumor	Improves targeting and procedural accuracy.
	Registration and alignment	European Society of Radiology [95]	Automatic/semiautomatic registration for multimodal fusion	CT, US, PET	Fusion-guided interventions	Improves lesion visualization and targeting.
Robotics	Endovascular applications	Beaman et al. [81]	Robotic actuation and image-guided navigation	CT, MRI, US, Fluoroscopy	Percutaneous and/or endovascular	Increases precision and standardization.
	Endovascular applications	Rueda et al. [82]	Robotic actuation and image-guided navigation	CT, MRI, US, Fluoroscopy	Percutaneous and/or endovascular	Increases precision and standardization.
	Percutaneous applications	Levy et al. [83]	Robotic actuation and image-guided navigation	CT, US	Percutaneous and/or Endovascular	Improves targeting and procedural accuracy. Motion compensation enhances safety.
	Percutaneous applications	Kettenbach et al. [84]	Robotic actuation and image-guided navigation	CT/MRI/US/Fluoro (varies)	Percutaneous and/or endovascular	Improves targeting and procedural accuracy.
	Percutaneous applications	Christou et al. [86]	Robotic actuation and image-guided navigation	CT, MRI, US	Biopsy, prostate	Improves targeting and procedural accuracy.
	Percutaneous applications	Chlorogiannis et al. [80]	Robotic actuation and image-guided navigation	CT/MRI/US/Fluoro (varies)	Percutaneous and/or endovascular	Increases precision and standardization.
	Percutaneous applications	Lanza et al. [61]	Robotic actuation and image-guided navigation	CT/MRI/US/Fluoro (varies)	Percutaneous and/or endovascular	Increases precision and standardization.
	Percutaneous applications	Berger et al. [85]	Robotic actuation and image-guided navigation	CT/MRI/US/Fluoro (varies)	Percutaneous and/or endovascular	Improves targeting and procedural accuracy.
	Percutaneous applications	Zheng et al. [87]	Robotic actuation and image-guided navigation	CT	Percutaneous and/or endovascular	Increases precision and standardization.
	Percutaneous applications	Barral et al. [10]	Robotic actuation and image-guided navigation	CT, MRI, US	Biopsy, prostate	Improves targeting and procedural accuracy.
VR/AR	Navigation and guidance	Gunduz et al. [93]	AI-assisted VR/AR guidance and trajectory suggestion	CT, US	Navigation and guidance	Shortens procedures and reduces radiation. Improves targeting and procedural accuracy.
	Navigation and guidance	Najafi et al. [94]	AI-assisted VR/AR guidance and trajectory suggestion	Fluoroscopy/US/CBCT (integrated)	Navigation and guidance	Improves workflow efficiency.
	Navigation and guidance	Rafii-Tari et al. [88]	AI-assisted VR/AR guidance and trajectory suggestion	Fluoroscopy/US/CBCT (integrated)	Navigation and guidance	Shortens procedures and reduces radiation. Improves targeting and procedural accuracy.
	Navigation and guidance	Allaqaband et al. [91]	AI-assisted VR/AR guidance and trajectory suggestion	CT, US	Navigation and guidance	Improves spatial orientation and safety.
	Navigation and guidance	Rao et al. [92]	AI-assisted VR/AR guidance and trajectory suggestion	CT	Embolization (TACE/TAE), peripheral revascularization	Improves spatial orientation and safety.
	Navigation and guidance	Alderliesten et al. [90]	AI-assisted VR/AR guidance and trajectory suggestion	CT, US	Navigation and guidance	Improves spatial orientation and safety.
	Navigation and guidance	Mendes Pereira et al. [89]	AI-assisted VR/AR guidance and trajectory suggestion	CT, US	Navigation and guidance	Improves spatial orientation and safety.

**Table 6:** Post-procedural applications of AI in IR.

Application Category	Author	Methodology	Imaging Modality	Key Findings
Tumor-related	Abajian et al. [3]	Random Forest	MRI	Combines MRI signal, contrast enhancement, and clinical parameters to classify TACE response; promising predictive performance
	Moon et al. [24]	Quantitative analysis (4D flow)	4D Flow MRI	Quantitative flow data predicts complete response after TACE in cirrhotic HCC patients
	Daye et al. [104]	ML integrating clinical + radiomic data	Pre-treatment CT	Predicts local tumor progression and survival after percutaneous thermal ablation (adrenal metastases) with high accuracy
Vascular	Li et al. [43]	ML	Clinical and imaging data	Predicts IVC filter complications by integrating clinical, anatomical, and device-related variables; supports personalized follow-up
	Charalambous et al. [103]	SVM	Post-EVAR CT angiography	Detects aggressive type II endoleaks associated with aneurysmal sac expansion; supports personalized surveillance
Procedural outcomes	Sinha et al. [105]	ML	CT, procedural imaging	Predicts pneumothorax post-CT biopsy, in-hospital mortality post-TIPS, prolonged hospital stay post-uterine artery embolization
General overview	Iezzi et al. [1]	Narrative Review of AI approaches	Multiple imaging modalities	Summarizes AI applications in IR across pre-, intra-, and post-procedural settings; highlights opportunities and future challenges
	Seah et al. [102]	ML, DL	Post-procedural imaging	Enhances workflow, reduces inter-observer variability, improves post-procedural assessment accuracy, quantifies treatment response, supports prognostic evaluation

## Data Availability

The data presented in this study are available on request from the corresponding author due to privacy restrictions.
